# Supplementary figures and images for: GFPrint™: A machine learning tool for transforming genetic data into clinical insights
Source: PLoS One. 2024 Nov 27;19(11):e0311370. doi: 10.1371/journal.pone.0311370 (PMC11602062; doi:10.1371/journal.pone.0311370)

**S1 Fig:** Diagram showing the workflow utilized by GFPrint™

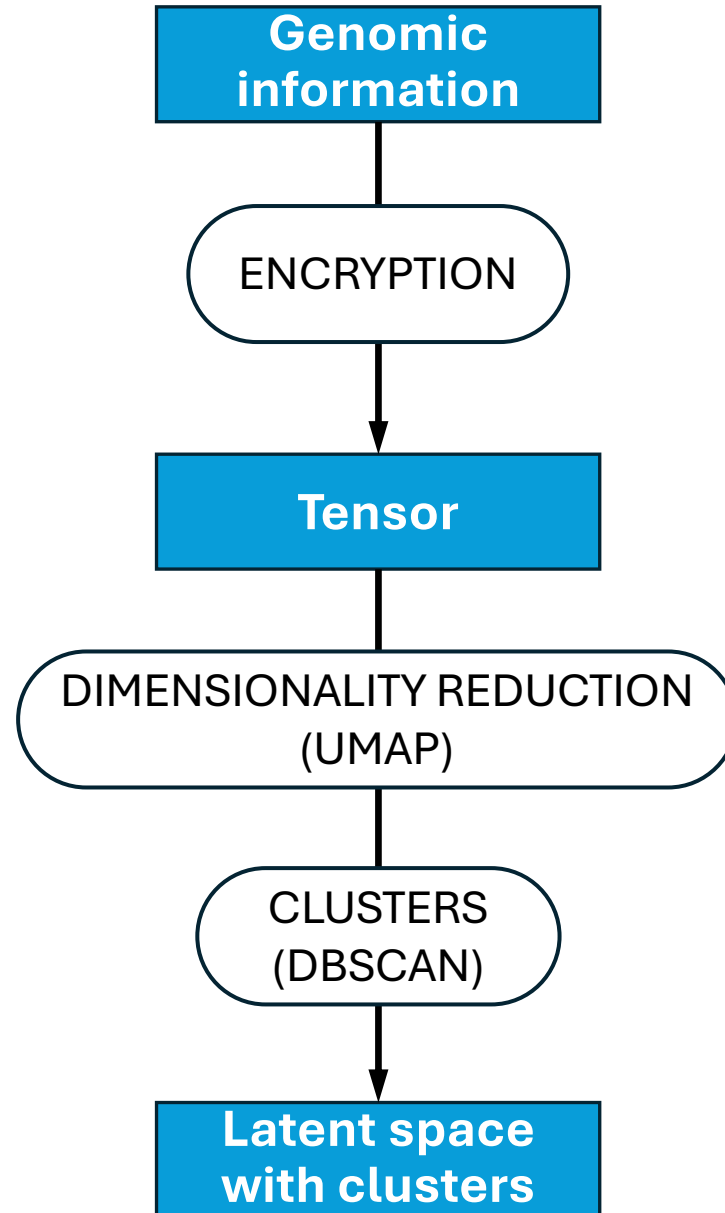

Supplement: S1 Fig — (PDF) [file pone.0311370.s001.pdf]
