# Supplementary material for: GFPrint™: A machine learning tool for transforming genetic data into clinical insights
Source: PLoS One. 2024 Nov 27;19(11):e0311370. doi: 10.1371/journal.pone.0311370 (PMC11602062; doi:10.1371/journal.pone.0311370)
Supplement: S1 Table — NOS, not otherwise specified. (PDF) [file pone.0311370.s002.pdf]

S1 Table: Distribution of the 145 tumor histotypes found in the TCGA dataset along the 25 cancer groups created according to the primary diagnosis described there.

NOS, not otherwise specified.

Cancer group	Histotype	Primary diagnosis
Adrenal	Adrenal gland, NOS	Ganglioneuroblastoma
	Adrenal gland, NOS	Neuroblastoma, NOS
	Adrenal gland, NOS	Paraganglioma, malignant
	Adrenal gland, NOS	Pheochromocytoma, malignant
	Adrenal gland, NOS	Pheochromocytoma, NOS
	Cortex of adrenal gland	Adrenal cortical carcinoma
	Cortex of adrenal gland	Ganglioneuroblastoma
	Cortex of adrenal gland	Neuroblastoma, NOS
	Cortex of adrenal gland	Pheochromocytoma, NOS
	Medulla of adrenal gland	Neuroblastoma, NOS
	Medulla of adrenal gland	Pheochromocytoma, malignant
	Retroperitoneum	Paraganglioma, NOS
	Retroperitoneum	Pheochromocytoma, NOS
Bone	Bone, NOS	Ewing sarcoma
	Bone, NOS	Not Reported
	Bone, NOS	Undifferentiated sarcoma
	Short bones of lower limb and associated joints	Leiomyosarcoma, NOS
Breast	Breast, NOS	Apocrine adenocarcinoma
	Breast, NOS	Cribriform carcinoma, NOS
	Breast, NOS	Infiltrating duct and lobular carcinoma
	Breast, NOS	Infiltrating duct carcinoma, NOS
	Breast, NOS	Infiltrating duct mixed with other types of carcinoma
	Breast, NOS	Infiltrating lobular mixed with other types of carcinoma
	Breast, NOS	Intraductal micropapillary carcinoma
	Breast, NOS	Intraductal papillary adenocarcinoma with invasion
	Breast, NOS	Large cell neuroendocrine carcinoma
	Breast, NOS	Lobular carcinoma, NOS
	Breast, NOS	Medullary carcinoma, NOS
	Breast, NOS	Metaplastic carcinoma, NOS
	Breast, NOS	Mucinous adenocarcinoma
	Breast, NOS	Paget disease and infiltrating duct carcinoma of breast
	Breast, NOS	Phyllodes tumor, malignant
	Breast, NOS	Tubular adenocarcinoma
	Lower-inner quadrant of breast	Infiltrating duct carcinoma, NOS
	Lower-inner quadrant of breast	Infiltrating duct mixed with other types of carcinoma
	Lower-outer quadrant of breast	Infiltrating duct carcinoma, NOS
	Overlapping lesion of breast	Infiltrating duct carcinoma, NOS
	Overlapping lesion of breast	Pleomorphic carcinoma

Cancer group	Histotype	Primary diagnosis
	Upper-inner quadrant of breast	Infiltrating duct carcinoma, NOS
	Upper-inner quadrant of breast	Infiltrating lobular mixed with other types of carcinoma
	Upper-outer quadrant of breast	Infiltrating duct carcinoma, NOS
CNS	Brain, NOS	Astrocytoma, anaplastic
	Brain, NOS	Astrocytoma, NOS
	Brain, NOS	Glioblastoma
	Brain, NOS	Gliosarcoma
	Brain, NOS	Mixed glioma
	Brain, NOS	Oligodendroglioma, anaplastic
	Brain, NOS	Oligodendroglioma, NOS
	Cerebrum	Astrocytoma, anaplastic
	Cerebrum	Astrocytoma, NOS
	Cerebrum	Mixed glioma
	Cerebrum	Oligodendroglioma, anaplastic
	Cerebrum	Oligodendroglioma, NOS
	Frontal lobe	Astrocytoma, anaplastic
	Frontal lobe	Astrocytoma, NOS
	Frontal lobe	Glioblastoma
	Frontal lobe	Mixed glioma
	Frontal lobe	Oligodendroglioma, anaplastic
	Occipital lobe	Astrocytoma, anaplastic
	Occipital lobe	Glioblastoma
	Overlapping lesion of brain	Glioblastoma
	Parietal lobe	Glioblastoma
	Parietal lobe	Mixed glioma
	Temporal lobe	Astrocytoma, anaplastic
	Temporal lobe	Glioblastoma
	Temporal lobe	Mixed glioma
	Temporal lobe	Oligodendroglioma, NOS
Connective and soft tissue	Connective, subcutaneous and other soft tissues of abdomen	Adenocarcinoma, NOS
	Connective, subcutaneous and other soft tissues of abdomen	Extra-adrenal paraganglioma, NOS
	Connective, subcutaneous and other soft tissues of abdomen	Leiomyosarcoma, NOS
	Connective, subcutaneous and other soft tissues of head, face, and neck	Malignant peripheral nerve sheath tumor
	Connective, subcutaneous and other soft tissues of head, face, and neck	Pleomorphic liposarcoma
	Connective, subcutaneous and other soft tissues of head, face, and neck	Synovial sarcoma, spindle cell
	Connective, subcutaneous and other soft tissues of head, face, and neck	Undifferentiated sarcoma
	Connective, subcutaneous and other soft tissues of lower limb and hip	Dedifferentiated liposarcoma
	Connective, subcutaneous and other soft tissues of lower limb and hip	Fibromyxosarcoma
	Connective, subcutaneous and other soft tissues of lower limb and hip	Giant cell sarcoma

Cancer group	Histotype	Primary diagnosis
	Connective, subcutaneous and other soft tissues of lower limb and hip	Leiomyosarcoma, NOS
	Connective, subcutaneous and other soft tissues of lower limb and hip	Malignant fibrous histiocytoma
	Connective, subcutaneous and other soft tissues of lower limb and hip	Malignant peripheral nerve sheath tumor
	Connective, subcutaneous and other soft tissues of lower limb and hip	Synovial sarcoma, biphasic
	Connective, subcutaneous and other soft tissues of lower limb and hip	Synovial sarcoma, spindle cell
	Connective, subcutaneous and other soft tissues of lower limb and hip	Undifferentiated sarcoma
	Connective, subcutaneous and other soft tissues of pelvis	Dedifferentiated liposarcoma
	Connective, subcutaneous and other soft tissues of pelvis	Extra-adrenal paraganglioma, NOS
	Connective, subcutaneous and other soft tissues of pelvis	Fibromyxosarcoma
	Connective, subcutaneous and other soft tissues of pelvis	Leiomyosarcoma, NOS
	Connective, subcutaneous and other soft tissues of pelvis	Malignant fibrous histiocytoma
	Connective, subcutaneous and other soft tissues of pelvis	Undifferentiated sarcoma
	Connective, subcutaneous and other soft tissues of thorax	Aggressive fibromatosis
	Connective, subcutaneous and other soft tissues of thorax	Dedifferentiated liposarcoma
	Connective, subcutaneous and other soft tissues of thorax	Extra-adrenal paraganglioma, NOS
	Connective, subcutaneous and other soft tissues of thorax	Fibromyxosarcoma
	Connective, subcutaneous and other soft tissues of thorax	Leiomyosarcoma, NOS
	Connective, subcutaneous and other soft tissues of thorax	Malignant fibrous histiocytoma
	Connective, subcutaneous and other soft tissues of thorax	Malignant peripheral nerve sheath tumor
	Connective, subcutaneous and other soft tissues of thorax	Paraganglioma, NOS
	Connective, subcutaneous and other soft tissues of thorax	Synovial sarcoma, NOS
	Connective, subcutaneous and other soft tissues of thorax	Synovial sarcoma, spindle cell
	Connective, subcutaneous and other soft tissues of thorax	Undifferentiated sarcoma
	Connective, subcutaneous and other soft tissues of trunk, NOS	Extra-adrenal paraganglioma, NOS
	Connective, subcutaneous and other soft tissues of trunk, NOS	Fibromyxosarcoma
	Connective, subcutaneous and other soft tissues of trunk, NOS	Leiomyosarcoma, NOS
	Connective, subcutaneous and other soft tissues of trunk, NOS	Malignant fibrous histiocytoma
	Connective, subcutaneous and other soft tissues of trunk, NOS	Malignant peripheral nerve sheath tumor
	Connective, subcutaneous and other soft tissues of trunk, NOS	Synovial sarcoma, biphasic
	Connective, subcutaneous and other soft tissues of trunk, NOS	Undifferentiated sarcoma
	Connective, subcutaneous and other soft tissues of upper limb and shoulder	Dedifferentiated liposarcoma

Cancer group	Histotype	Primary diagnosis
	Connective, subcutaneous and other soft tissues of upper limb and shoulder	Leiomyosarcoma, NOS
	Connective, subcutaneous and other soft tissues of upper limb and shoulder	Synovial sarcoma, NOS
	Connective, subcutaneous and other soft tissues of upper limb and shoulder	Undifferentiated sarcoma
	Connective, subcutaneous and other soft tissues, NOS	Alveolar rhabdomyosarcoma
	Connective, subcutaneous and other soft tissues, NOS	Leiomyosarcoma, NOS
	Connective, subcutaneous and other soft tissues, NOS	Undifferentiated sarcoma
	Overlapping lesion of connective, subcutaneous and other soft tissues	Leiomyosarcoma, NOS
	Peripheral nerves and autonomic nervous system of upper limb and shoulder	Malignant peripheral nerve sheath tumor
	Retroperitoneum	Malignant peripheral nerve sheath tumor
	Spinal meninges	Malignant peripheral nerve sheath tumor
Esophagus	Esophagus, NOS	Adenocarcinoma, NOS
	Esophagus, NOS	Squamous cell carcinoma, keratinizing, NOS
	Esophagus, NOS	Squamous cell carcinoma, NOS
	Lower third of esophagus	Adenocarcinoma, NOS
	Lower third of esophagus	Basaloid squamous cell carcinoma
	Lower third of esophagus	Mucinous adenocarcinoma
	Lower third of esophagus	Squamous cell carcinoma, keratinizing, NOS
	Lower third of esophagus	Squamous cell carcinoma, NOS
	Lower third of esophagus	Tubular adenocarcinoma
	Middle third of esophagus	Adenocarcinoma, NOS
	Middle third of esophagus	Squamous cell carcinoma, NOS
	Thoracic esophagus	Adenocarcinoma, NOS
	Thoracic esophagus	Squamous cell carcinoma, keratinizing, NOS
	Upper third of esophagus	Squamous cell carcinoma, NOS
Head and neck	Anterior floor of mouth	Squamous cell carcinoma, keratinizing, NOS
	Anterior floor of mouth	Squamous cell carcinoma, NOS
	Base of tongue, NOS	Basaloid squamous cell carcinoma
	Base of tongue, NOS	Squamous cell carcinoma, keratinizing, NOS
	Base of tongue, NOS	Squamous cell carcinoma, large cell, nonkeratinizing, NOS
	Base of tongue, NOS	Squamous cell carcinoma, NOS
	Border of tongue	Squamous cell carcinoma, NOS
	Cheek mucosa	Basaloid squamous cell carcinoma
	Cheek mucosa	Squamous cell carcinoma, keratinizing, NOS
	Cheek mucosa	Squamous cell carcinoma, NOS
	Floor of mouth, NOS	Basaloid squamous cell carcinoma
	Floor of mouth, NOS	Squamous cell carcinoma, keratinizing, NOS
	Floor of mouth, NOS	Squamous cell carcinoma, NOS

Cancer group	Histotype	Primary diagnosis
	Gum, NOS	Squamous cell carcinoma, keratinizing, NOS
	Gum, NOS	Squamous cell carcinoma, NOS
	Hard palate	Squamous cell carcinoma, NOS
	Head, face or neck, NOS	Extra-adrenal paraganglioma, malignant
	Head, face or neck, NOS	Squamous cell carcinoma, NOS
	Hypopharynx, NOS	Squamous cell carcinoma, large cell, nonkeratinizing, NOS
	Hypopharynx, NOS	Squamous cell carcinoma, NOS
	Larynx, NOS	Basaloid squamous cell carcinoma
	Larynx, NOS	Squamous cell carcinoma, keratinizing, NOS
	Larynx, NOS	Squamous cell carcinoma, large cell, nonkeratinizing, NOS
	Larynx, NOS	Squamous cell carcinoma, NOS
	Lip, NOS	Squamous cell carcinoma, keratinizing, NOS
	Lip, NOS	Squamous cell carcinoma, NOS
	Lower gum	Squamous cell carcinoma, NOS
	Mandible	Squamous cell carcinoma, NOS
	Mouth, NOS	Squamous cell carcinoma, keratinizing, NOS
	Mouth, NOS	Squamous cell carcinoma, NOS
	Oropharynx, NOS	Squamous cell carcinoma, keratinizing, NOS
	Oropharynx, NOS	Squamous cell carcinoma, NOS
	Oropharynx, NOS	Squamous cell carcinoma, spindle cell
	Overlapping lesion of lip, oral cavity and pharynx	Squamous cell carcinoma, NOS
	Palate, NOS	Squamous cell carcinoma, keratinizing, NOS
	Pharynx, NOS	Squamous cell carcinoma, NOS
	Posterior wall of oropharynx	Squamous cell carcinoma, keratinizing, NOS
	Supraglottis	Squamous cell carcinoma, NOS
	Tongue, NOS	Leiomyosarcoma, NOS
	Tongue, NOS	Squamous cell carcinoma, keratinizing, NOS
	Tongue, NOS	Squamous cell carcinoma, NOS
	Tonsil, NOS	Basaloid squamous cell carcinoma
	Tonsil, NOS	Squamous cell carcinoma, keratinizing, NOS
	Tonsil, NOS	Squamous cell carcinoma, large cell, nonkeratinizing, NOS
	Tonsil, NOS	Squamous cell carcinoma, NOS
	Upper Gum	Squamous cell carcinoma, NOS
Hematological	Bone marrow	Acute myeloid leukemia, NOS
	Bone marrow	B lymphoblastic leukemia/lymphoma, NOS
	Bone marrow	Leukemia, NOS
	Bone marrow	Mixed phenotype acute leukemia with t(v;11q23); MLL rearranged
	Bone marrow	Mixed phenotype acute leukemia, B/myeloid, NOS

Cancer group	Histotype	Primary diagnosis
	Bone marrow	Mixed phenotype acute leukemia, T/myeloid, NOS
	Bone marrow	Multiple myeloma
	Bone marrow	Not Reported
	Bone marrow	Undifferentiated leukaemia
	Bones of skull and face and associated joints	Malignant lymphoma, large B-cell, diffuse, NOS
	Brain stem	Malignant lymphoma, large B-cell, diffuse, NOS
	Breast, NOS	Malignant lymphoma, large B-cell, diffuse, NOS
	Cerebellum, NOS	Malignant lymphoma, large B-cell, diffuse, NOS
	Connective, subcutaneous and other soft tissues of head, face, and neck	Malignant lymphoma, large B-cell, diffuse, NOS
	Connective, subcutaneous and other soft tissues, NOS	Malignant lymphoma, large B-cell, diffuse, NOS
	Intra-abdominal lymph nodes	Malignant lymphoma, large B-cell, diffuse, NOS
	Intrathoracic lymph nodes	Malignant lymphoma, large B-cell, diffuse, NOS
	Lymph nodes of axilla or arm	Malignant lymphoma, large B-cell, diffuse, NOS
	Lymph nodes of head, face and neck	Malignant lymphoma, large B-cell, diffuse, NOS
	Lymph nodes of inguinal region or leg	Malignant lymphoma, large B-cell, diffuse, NOS
	Specified parts of peritoneum	Malignant lymphoma, large B-cell, diffuse, NOS
	Submandibular gland	Malignant lymphoma, large B-cell, diffuse, NOS
	Thyroid gland	Malignant lymphoma, large B-cell, diffuse, NOS
Kidney	Kidney, NOS	Clear cell adenocarcinoma, NOS
	Kidney, NOS	Neuroblastoma, NOS
	Kidney, NOS	Papillary adenocarcinoma, NOS
	Kidney, NOS	Renal cell carcinoma, chromophobe type
	Kidney, NOS	Renal cell carcinoma, NOS
	Kidney, NOS	Synovial sarcoma, spindle cell
	Kidney, NOS	Wilms tumor
Liver and biliary tract	Ampulla of Vater	Adenocarcinoma, NOS
	Extrahepatic bile duct	Adenocarcinoma, NOS
	Extrahepatic bile duct	Cholangiocarcinoma
	Gallbladder	Cholangiocarcinoma
	Intrahepatic bile duct	Cholangiocarcinoma
	Liver	Cholangiocarcinoma
	Liver	Clear cell adenocarcinoma, NOS
	Liver	Combined hepatocellular carcinoma and cholangiocarcinoma
	Liver	Hepatocellular carcinoma, clear cell type
	Liver	Hepatocellular carcinoma, fibrolamellar
	Liver	Hepatocellular carcinoma, NOS
	Liver	Hepatocellular carcinoma, spindle cell variant

Cancer group	Histotype	Primary diagnosis
Lung and pleura	Lower lobe, lung	Acinar cell carcinoma
	Lower lobe, lung	Adenocarcinoma with mixed subtypes
	Lower lobe, lung	Adenocarcinoma, NOS
	Lower lobe, lung	Basaloid squamous cell carcinoma
	Lower lobe, lung	Bronchio-alveolar carcinoma, mucinous
	Lower lobe, lung	Bronchiolo-alveolar adenocarcinoma, NOS
	Lower lobe, lung	Bronchiolo-alveolar carcinoma, non-mucinous
	Lower lobe, lung	Clear cell adenocarcinoma, NOS
	Lower lobe, lung	Micropapillary carcinoma, NOS
	Lower lobe, lung	Mucinous adenocarcinoma
	Lower lobe, lung	Papillary adenocarcinoma, NOS
	Lower lobe, lung	Papillary squamous cell carcinoma
	Lower lobe, lung	Solid carcinoma, NOS
	Lower lobe, lung	Squamous cell carcinoma, keratinizing, NOS
	Lower lobe, lung	Squamous cell carcinoma, large cell, nonkeratinizing, NOS
	Lower lobe, lung	Squamous cell carcinoma, NOS
	Lung, NOS	Adenocarcinoma with mixed subtypes
	Lung, NOS	Adenocarcinoma, NOS
	Lung, NOS	Papillary adenocarcinoma, NOS
	Lung, NOS	Squamous cell carcinoma, keratinizing, NOS
	Lung, NOS	Squamous cell carcinoma, NOS
	Main bronchus	Adenocarcinoma, NOS
	Main bronchus	Squamous cell carcinoma, NOS
	Middle lobe, lung	Adenocarcinoma with mixed subtypes
	Middle lobe, lung	Adenocarcinoma, NOS
	Middle lobe, lung	Papillary adenocarcinoma, NOS
	Middle lobe, lung	Squamous cell carcinoma, keratinizing, NOS
	Middle lobe, lung	Squamous cell carcinoma, NOS
	Middle lobe, lung	Squamous cell carcinoma, small cell, nonkeratinizing
	Overlapping lesion of lung	Adenocarcinoma with mixed subtypes
	Overlapping lesion of lung	Bronchiolo-alveolar carcinoma, non-mucinous
	Overlapping lesion of lung	Papillary adenocarcinoma, NOS
	Overlapping lesion of lung	Squamous cell carcinoma, NOS
	Pleura, NOS	Epithelioid mesothelioma, malignant
	Pleura, NOS	Mesothelioma, biphasic, malignant
	Pleura, NOS	Mesothelioma, malignant
	Upper lobe, lung	Acinar cell carcinoma
	Upper lobe, lung	Adenocarcinoma with mixed subtypes
	Upper lobe, lung	Adenocarcinoma, NOS
	Upper lobe, lung	Basaloid squamous cell carcinoma
	Upper lobe, lung	Bronchio-alveolar carcinoma, mucinous

Cancer group	Histotype	Primary diagnosis
	Upper lobe, lung	Bronchiolo-alveolar adenocarcinoma, NOS
	Upper lobe, lung	Bronchiolo-alveolar carcinoma, non-mucinous
	Upper lobe, lung	Clear cell adenocarcinoma, NOS
	Upper lobe, lung	Micropapillary carcinoma, NOS
	Upper lobe, lung	Mucinous adenocarcinoma
	Upper lobe, lung	Papillary adenocarcinoma, NOS
	Upper lobe, lung	Papillary squamous cell carcinoma
	Upper lobe, lung	Signet ring cell carcinoma
	Upper lobe, lung	Solid carcinoma, NOS
	Upper lobe, lung	Squamous cell carcinoma, keratinizing, NOS
	Upper lobe, lung	Squamous cell carcinoma, large cell, nonkeratinizing, NOS
	Upper lobe, lung	Squamous cell carcinoma, NOS
Mediastinum	Anterior mediastinum	Malignant lymphoma, large B-cell, diffuse, NOS
	Anterior mediastinum	Thymic carcinoma, NOS
	Anterior mediastinum	Thymoma, type A, malignant
	Anterior mediastinum	Thymoma, type AB, malignant
	Anterior mediastinum	Thymoma, type AB, NOS
	Anterior mediastinum	Thymoma, type B1, malignant
	Anterior mediastinum	Thymoma, type B2, malignant
	Anterior mediastinum	Thymoma, type B2, NOS
	Anterior mediastinum	Thymoma, type B3, malignant
	Mediastinum, NOS	Extra-adrenal paraganglioma, malignant
	Mediastinum, NOS	Neuroblastoma, NOS
	Mediastinum, NOS	Thymoma, type A, malignant
	Mediastinum, NOS	Thymoma, type B1, malignant
	Mediastinum, NOS	Thymoma, type B2, malignant
	Posterior mediastinum	Neuroblastoma, NOS
	Thymus	Thymic carcinoma, NOS
	Thymus	Thymoma, type A, malignant
	Thymus	Thymoma, type A, NOS
	Thymus	Thymoma, type AB, malignant
	Thymus	Thymoma, type AB, NOS
	Thymus	Thymoma, type B1, malignant
	Thymus	Thymoma, type B1, NOS
	Thymus	Thymoma, type B2, malignant
	Thymus	Thymoma, type B2, NOS
	Thymus	Thymoma, type B3, malignant
Neuroendocrine	Abdomen, NOS	Ganglioneuroblastoma
	Abdomen, NOS	Neuroblastoma, NOS
	Aortic body and other paraganglia	Pheochromocytoma, malignant
	Bone marrow	Neuroblastoma, NOS
	Bones of skull and face and associated joints	Neuroblastoma, NOS

Cancer group	Histotype	Primary diagnosis
	Connective, subcutaneous and other soft tissues of abdomen	Ganglioneuroblastoma
	Connective, subcutaneous and other soft tissues of abdomen	Neuroblastoma, NOS
	Connective, subcutaneous and other soft tissues of abdomen	Paraganglioma, NOS
	Head, face or neck, NOS	Neuroblastoma, NOS
	Heart	Extra-adrenal paraganglioma, NOS
	Intra-abdominal lymph nodes	Neuroblastoma, NOS
	Liver	Neuroblastoma, NOS
	Long bones of lower limb and associated joints	Neuroblastoma, NOS
	Nervous system, NOS	Paraganglioma, malignant
	Pelvic lymph nodes	Ganglioneuroblastoma
	Peripheral nerves and autonomic nervous system of abdomen	Neuroblastoma, NOS
	Peripheral nerves and autonomic nervous system of thorax	Neuroblastoma, NOS
	Spinal meninges	Neuroblastoma, NOS
	Thorax, NOS	Extra-adrenal paraganglioma, NOS
	Thorax, NOS	Neuroblastoma, NOS
	Unknown	Ganglioneuroblastoma
	Unknown	Neuroblastoma, NOS
Ovary	Ovary	Cystadenocarcinoma, NOS
	Ovary	Leiomyosarcoma, NOS
	Ovary	Papillary serous cystadenocarcinoma
	Ovary	Serous cystadenocarcinoma, NOS
Pancreas	Body of pancreas	Adenocarcinoma, NOS
	Body of pancreas	Infiltrating duct carcinoma, NOS
	Body of pancreas	Neuroendocrine carcinoma, NOS
	Head of pancreas	Adenocarcinoma with mixed subtypes
	Head of pancreas	Adenocarcinoma, NOS
	Head of pancreas	Infiltrating duct carcinoma, NOS
	Head of pancreas	Mucinous adenocarcinoma
	Head of pancreas	Neuroendocrine carcinoma, NOS
	Overlapping lesion of pancreas	Adenocarcinoma, NOS
	Overlapping lesion of pancreas	Infiltrating duct carcinoma, NOS
	Pancreas, NOS	Adenocarcinoma, NOS
	Pancreas, NOS	Infiltrating duct carcinoma, NOS
	Pancreas, NOS	Neuroendocrine carcinoma, NOS
	Tail of pancreas	Adenocarcinoma, NOS
	Tail of pancreas	Carcinoma, undifferentiated, NOS
	Tail of pancreas	Infiltrating duct carcinoma, NOS
	Tail of pancreas	Neuroendocrine carcinoma, NOS
	Unknown primary site	Mucinous adenocarcinoma
Prostate	Prostate gland	Adenocarcinoma with mixed subtypes
	Prostate gland	Adenocarcinoma, NOS

Cancer group	Histotype	Primary diagnosis
	Prostate gland	Infiltrating duct carcinoma, NOS
	Prostate gland	Mucinous adenocarcinoma
Retroperitoneum	Overlapping lesion of retroperitoneum and peritoneum	Neuroblastoma, NOS
	Retroperitoneum	Abdominal fibromatosis
	Retroperitoneum	Dedifferentiated liposarcoma
	Retroperitoneum	Extra-adrenal paraganglioma, malignant
	Retroperitoneum	Extra-adrenal paraganglioma, NOS
	Retroperitoneum	Fibromyxosarcoma
	Retroperitoneum	Ganglioneuroblastoma
	Retroperitoneum	Giant cell sarcoma
	Retroperitoneum	Leiomyosarcoma, NOS
	Retroperitoneum	Liposarcoma, well differentiated
	Retroperitoneum	Myxoid leiomyosarcoma
	Retroperitoneum	Neuroblastoma, NOS
	Retroperitoneum	Undifferentiated sarcoma
	Specified parts of peritoneum	Dedifferentiated liposarcoma
Skin	Choroid	Epithelioid cell melanoma
	Choroid	Malignant melanoma, NOS
	Choroid	Mixed epithelioid and spindle cell melanoma
	Choroid	Spindle cell melanoma, NOS
	Choroid	Spindle cell melanoma, type B
	Ciliary body	Epithelioid cell melanoma
	Ciliary body	Mixed epithelioid and spindle cell melanoma
	Ciliary body	Spindle cell melanoma, NOS
	Overlapping lesion of eye and adnexa	Mixed epithelioid and spindle cell melanoma
	Overlapping lesion of eye and adnexa	Spindle cell melanoma, NOS
	Overlapping lesion of eye and adnexa	Spindle cell melanoma, type B
	Skin, NOS	Acral lentiginous melanoma, malignant
	Skin, NOS	Amelanotic melanoma
	Skin, NOS	Desmoplastic melanoma, malignant
	Skin, NOS	Epithelioid cell melanoma
	Skin, NOS	Lentigo maligna melanoma
	Skin, NOS	Malignant melanoma, NOS
	Skin, NOS	Mixed epithelioid and spindle cell melanoma
	Skin, NOS	Nodular melanoma
	Skin, NOS	Spindle cell melanoma, NOS
	Skin, NOS	Spindle cell sarcoma
	Skin, NOS	Superficial spreading melanoma
Small & large bowel	Ascending colon	Adenocarcinoma, NOS
	Ascending colon	Adenosquamous carcinoma
	Ascending colon	Mucinous adenocarcinoma

Cancer group	Histotype	Primary diagnosis
	Ascending colon	Papillary adenocarcinoma, NOS
	Cecum	Adenocarcinoma, NOS
	Cecum	Mucinous adenocarcinoma
	Colon, NOS	Adenocarcinoma with neuroendocrine differentiation
	Colon, NOS	Adenocarcinoma, NOS
	Colon, NOS	Malignant lymphoma, large B-cell, diffuse, NOS
	Colon, NOS	Mucinous adenocarcinoma
	Descending colon	Adenocarcinoma, NOS
	Descending colon	Dedifferentiated liposarcoma
	Descending colon	Mucinous adenocarcinoma
	Hepatic flexure of colon	Adenocarcinoma, NOS
	Hepatic flexure of colon	Mucinous adenocarcinoma
	Jejunum	Malignant lymphoma, large B-cell, diffuse, NOS
	Rectosigmoid junction	Adenocarcinoma, NOS
	Rectosigmoid junction	Mucinous adenocarcinoma
	Rectum, NOS	Adenocarcinoma in tubolovillous adenoma
	Rectum, NOS	Adenocarcinoma with mixed subtypes
	Rectum, NOS	Adenocarcinoma, NOS
	Rectum, NOS	Mucinous adenocarcinoma
	Rectum, NOS	Tubular adenocarcinoma
	Sigmoid colon	Adenocarcinoma, NOS
	Sigmoid colon	Carcinoma, NOS
	Sigmoid colon	Mucinous adenocarcinoma
	Sigmoid colon	Papillary adenocarcinoma, NOS
	Small intestine, NOS	Malignant lymphoma, large B-cell, diffuse, NOS
	Splenic flexure of colon	Adenocarcinoma, NOS
	Splenic flexure of colon	Mucinous adenocarcinoma
	Transverse colon	Adenocarcinoma, NOS
	Transverse colon	Mucinous adenocarcinoma
Spermatic cord	Spermatic cord	Dedifferentiated liposarcoma
Stomach	Body of stomach	Adenocarcinoma, intestinal type
	Body of stomach	Adenocarcinoma, NOS
	Body of stomach	Carcinoma, diffuse type
	Body of stomach	Mucinous adenocarcinoma
	Body of stomach	Papillary adenocarcinoma, NOS
	Body of stomach	Signet ring cell carcinoma
	Body of stomach	Tubular adenocarcinoma
	Cardia, NOS	Adenocarcinoma, intestinal type
	Cardia, NOS	Adenocarcinoma, NOS
	Cardia, NOS	Carcinoma, diffuse type
	Cardia, NOS	Mucinous adenocarcinoma
	Cardia, NOS	Papillary adenocarcinoma, NOS

Cancer group	Histotype	Primary diagnosis
	Cardia, NOS	Signet ring cell carcinoma
	Cardia, NOS	Squamous cell carcinoma, NOS
	Cardia, NOS	Tubular adenocarcinoma
	Fundus of stomach	Adenocarcinoma, intestinal type
	Fundus of stomach	Adenocarcinoma, NOS
	Fundus of stomach	Carcinoma, diffuse type
	Fundus of stomach	Mucinous adenocarcinoma
	Fundus of stomach	Papillary adenocarcinoma, NOS
	Fundus of stomach	Tubular adenocarcinoma
	Gastric antrum	Adenocarcinoma, intestinal type
	Gastric antrum	Adenocarcinoma, NOS
	Gastric antrum	Carcinoma, diffuse type
	Gastric antrum	Mucinous adenocarcinoma
	Gastric antrum	Papillary adenocarcinoma, NOS
	Gastric antrum	Signet ring cell carcinoma
	Gastric antrum	Tubular adenocarcinoma
	Lesser curvature of stomach, NOS	Adenocarcinoma, NOS
	Stomach, NOS	Adenocarcinoma with mixed subtypes
	Stomach, NOS	Adenocarcinoma, intestinal type
	Stomach, NOS	Adenocarcinoma, NOS
	Stomach, NOS	Carcinoma, diffuse type
	Stomach, NOS	Dedifferentiated liposarcoma
	Stomach, NOS	Leiomyosarcoma, NOS
	Stomach, NOS	Malignant lymphoma, large B-cell, diffuse, NOS
	Stomach, NOS	Mucinous adenocarcinoma
Testis	Testis, NOS	Embryonal carcinoma, NOS
	Testis, NOS	Malignant lymphoma, large B-cell, diffuse, NOS
	Testis, NOS	Mixed germ cell tumor
	Testis, NOS	Seminoma, NOS
	Testis, NOS	Teratocarcinoma
	Testis, NOS	Teratoma, benign
	Testis, NOS	Teratoma, malignant, NOS
	Testis, NOS	Yolk sac tumor
Thyroid	Thyroid gland	Carcinoma, NOS
	Thyroid gland	Follicular adenocarcinoma, NOS
	Thyroid gland	Follicular carcinoma, minimally invasive
	Thyroid gland	Nonencapsulated sclerosing carcinoma
	Thyroid gland	Oxyphilic adenocarcinoma
	Thyroid gland	Papillary adenocarcinoma, NOS
	Thyroid gland	Papillary carcinoma, columnar cell
	Thyroid gland	Papillary carcinoma, follicular variant
Urinary tract	Anterior wall of bladder	Papillary transitional cell carcinoma
	Anterior wall of bladder	Transitional cell carcinoma

Cancer group	Histotype	Primary diagnosis
	Bladder neck	Transitional cell carcinoma
	Bladder, NOS	Carcinoma, NOS
	Bladder, NOS	Papillary transitional cell carcinoma
	Bladder, NOS	Squamous cell carcinoma, NOS
	Bladder, NOS	Transitional cell carcinoma
	Dome of bladder	Papillary adenocarcinoma, NOS
	Dome of bladder	Transitional cell carcinoma
	Lateral wall of bladder	Papillary transitional cell carcinoma
	Lateral wall of bladder	Transitional cell carcinoma
	Posterior wall of bladder	Papillary transitional cell carcinoma
	Posterior wall of bladder	Transitional cell carcinoma
	Trigone of bladder	Papillary transitional cell carcinoma
	Trigone of bladder	Transitional cell carcinoma
	Ureteric orifice	Transitional cell carcinoma
Uterus	Cervix uteri	Adenocarcinoma, endocervical type
	Cervix uteri	Adenocarcinoma, NOS
	Cervix uteri	Adenosquamous carcinoma
	Cervix uteri	Basaloid squamous cell carcinoma
	Cervix uteri	Endometrioid adenocarcinoma, NOS
	Cervix uteri	Mucinous adenocarcinoma, endocervical type
	Cervix uteri	Papillary squamous cell carcinoma
	Cervix uteri	Squamous cell carcinoma, keratinizing, NOS
	Cervix uteri	Squamous cell carcinoma, large cell, nonkeratinizing, NOS
	Cervix uteri	Squamous cell carcinoma, NOS
	Corpus uteri	Carcinosarcoma, NOS
	Corpus uteri	Endometrioid adenocarcinoma, NOS
	Corpus uteri	Leiomyosarcoma, NOS
	Corpus uteri	Mullerian mixed tumor
	Endometrium	Adenocarcinoma, NOS
	Endometrium	Carcinoma, undifferentiated, NOS
	Endometrium	Clear cell adenocarcinoma, NOS
	Endometrium	Endometrioid adenocarcinoma, NOS
	Endometrium	Endometrioid adenocarcinoma, secretory variant
	Endometrium	Papillary serous cystadenocarcinoma
	Endometrium	Serous cystadenocarcinoma, NOS
	Endometrium	Serous surface papillary carcinoma
	Fundus uteri	Endometrioid adenocarcinoma, NOS
	Fundus uteri	Serous cystadenocarcinoma, NOS
	Isthmus uteri	Endometrioid adenocarcinoma, NOS
	Isthmus uteri	Serous cystadenocarcinoma, NOS
	Myometrium	Leiomyosarcoma, NOS
	Uterus, NOS	Carcinosarcoma, NOS

Cancer group	Histotype	Primary diagnosis
	Uterus, NOS	Leiomyosarcoma, NOS
	Uterus, NOS	Mesodermal mixed tumor
	Uterus, NOS	Mullerian mixed tumor
	Uterus, NOS	Myxoid leiomyosarcoma
