# Supplementary material for: GFPrint™: A machine learning tool for transforming genetic data into clinical insights
Source: PLoS One. 2024 Nov 27;19(11):e0311370. doi: 10.1371/journal.pone.0311370 (PMC11602062; doi:10.1371/journal.pone.0311370)
Supplement: S2 Table — Statistical significance was evaluated using the log-rank (Mantel-Cox) test. n, number of patients in the cluster; MOS, median OS (in years); CI95, 95% confidence interval; HR, hazard ratio; NR, not reachable. (PDF) [file pone.0311370.s003.pdf]

S2 Table: Median survival and hazard ratios for the Kaplan Meier curves shown in Fig 2.

Statistical significance was evaluated using the log-rank (Mantel-Cox) test. n, number of patients in the cluster; MOS, median OS (in years); CI95, 95% confidence interval; HR, hazard ratio; NR, not reachable.

Cancer group	Cluster 0			Cluster 1			Comparative		
	n	MOS	MS CI95	n	MOS	MS CI95	p-value	HR	HR CI95
Small & large bowel	30	3.2	1.49-NR	523	8.3	5.84-NR	.004	2.5	1.33-4.76
Lung & pleura	149	2.9	2.42-3.72	1079	4.4	3.69-4.87	.003	1.47	1.14-1.89
Urinary tract	30	1.6	0.97-NR	370	2.9	2.13-5.4	.045	1.67	1.01-2.78
Adrenal	218	NR	NR	111	4.8	3.85-7.79	.0002	2.13	1.43-3.23
CNS	187	4.1	2.8-12.08	784	2.0	1.77-2.2	.00003	1.75	1.33-2.27
Mediastinum	89	NR	NR	37	NR	7.97-NR	.036	3.45	1.09-11.11
