# Supplementary material for: GFPrint™: A machine learning tool for transforming genetic data into clinical insights
Source: PLoS One. 2024 Nov 27;19(11):e0311370. doi: 10.1371/journal.pone.0311370 (PMC11602062; doi:10.1371/journal.pone.0311370)
Supplement: S3 Table — Pathologies are defined according to either the tumor type or to the precise anatomic location. This definition only includes those pathologies for which a relevant number of patients were found in the TCGA dataset, and it is intended to be as inclusive as possible, hence overlapping criteria have been followed in some cases. Therefore, some patients may have been included in more than one pathology. (1) These pathologies are paraganglioma, ganglioneuroblastoma and neuroblastoma.(2) MPNST, malignant peripheral nerve sheath tumor.(3) aNSCLC, adenocarcinoma non-small cell lung cancer. (4) eNSCLC, epidermoid non-small cell lung cancer. (PDF) [file pone.0311370.s004.pdf]

**S3 Table: Pathologies included in the 24 selected cancer groups.**

Pathologies are defined according to either the tumor type or to the precise anatomic location. This definition only includes those pathologies for which a relevant number of patients were found in the TCGA dataset, and it is intended to be as inclusive as possible, hence overlapping criteria have been followed in some cases. Therefore, some patients may have been included in more than one pathology.

(1) These pathologies are paraganglioma, ganglioneuroblastoma and neuroblastoma

(2) MPNST, malignant peripheral nerve sheath tumor

(3) aNSCLC, adenocarcinoma non-small cell lung cancer

(4) eNSCLC, epidermoid non-small cell lung cancer

| Cancer group | Pathologies |
|---|---|
| Adrenal | Adrenal carcinoma |
| | Pheochromocytoma |
| | Neuroblastoma |
| | Adrenal cancer of neural origin (1) |
| | Adrenocortical carcinoma |
| | Adrenal medullary carcinoma |
| Bone | Osteosarcoma |
| Breast | Ductal breast carcinoma |
| | Lobular breast carcinoma |
| | Breast cancer - other |
| CNS | Anaplasic astrocytoma |
| | Non-anaplasic astrocytoma |
| | Glioblastoma |
| | Oligodendroglioma |
| | CNS - other |
| Connective & soft tissue | Sarcoma |
| | Histiocytoma |
| | MPNST (2) |
| Esophagus | Esophagous adenocarcinoma |
| | Esophagus squamous cell carcinoma |
| | Middle esophageal carcinoma |
| | Lower esophageal carcinoma |
| Head & neck | Mouth cancer |
| | Pharynx cancer |
| | Larynx cancer |
| | Tongue cancer |
| Hematological | Acute lymphocitic leukemia |
| | Acute myeloid leukemia |
| | Lymphoma |
| | Myeloma |
| Kidney | Clear cell renal cell carcinoma |
| | Chromophobe renal cell carcinoma |
| | Papilary renal cell carcinoma |

| Cancer group | Pathologies |
|---|---|
| | Nephroblastoma |
| | Kidney cancer - other |
| Liver and biliary tract | Liver adenocarcinoma |
| | Hepatocellular carcinoma |
| | Cholangiocarcinoma |
| Lung and pleura | Mesothelioma |
| | Lung adenocarcinoma (aNSCLC)[(3)] |
| | Squamous cell lung cancer (eNSCLC)[(4)] |
| Mediastinum | Mediastinal epithelial neoplasia |
| | Anterior mediastinum tumor |
| | Thymoma |
| Neuroendocrine | Neuroblastoma |
| | Other - neuroendocrine |
| Ovary | Ovarian cancer |
| Pancreas | Exocrine pancreatic cancer |
| | Pancreatic adenocarcinoma |
| | Pancreatic ductal adenocarcinoma |
| Retroperitoneum | Retroperitoneal sarcoma |
| Prostate | Prostate adenocarcinoma |
| Skin | Skin melanoma |
| | Ocular melanoma |
| Small & large bowel | Colorectal cancer |
| | Rectal cancer |
| | Colon cancer |
| | Right colon cancer |
| | Left colon cancer |
| | Colorectal adenocarcinoma |
| | Mucinous colorectal adenocarcinoma |
| Stomach | Gastric adenocarcinoma |
| | Gastric diffuse adenocarcinoma |
| | Intestinal type gastric adenocarcinoma |
| Testis | Testicular cancer |
| | Testicular germ cell cancer |
| Thyroid | Thyroid cancer |
| Urinary tract | Urothelial carcinoma |
| Uterus | Cervical cancer |
| | Endometrial cancer |
| | Cancer of the corpus uteri |
| | Uterine serous carcinoma |
| | Uterine squamous cell carcinoma |
| | Uterine adenocarcinoma |
| | Endometrial adenocarcinoma |
| | Mixed Mullerian tumor |
