# Supplementary material for: GFPrint™: A machine learning tool for transforming genetic data into clinical insights
Source: PLoS One. 2024 Nov 27;19(11):e0311370. doi: 10.1371/journal.pone.0311370 (PMC11602062; doi:10.1371/journal.pone.0311370)
Supplement: S4 Table — (PDF) [file pone.0311370.s005.pdf]

S4 Table: List of genes harboring mutations exclusively found in non-metastatic CRC patients included in cluster 0

Gene name
OR5C1
LOC107985532
MAP7D2
SNORD116-29
NMS
GOLGA7
SNORD3B-1
LINC01098
NKX2-4
MIR17HG
IGF2-AS
GIP
CBWD6
MIR889
CCDC163
