# Supplementary material for: GFPrint™: A machine learning tool for transforming genetic data into clinical insights
Source: PLoS One. 2024 Nov 27;19(11):e0311370. doi: 10.1371/journal.pone.0311370 (PMC11602062; doi:10.1371/journal.pone.0311370)
Supplement: S5 Table — (PDF) [file pone.0311370.s006.pdf]

S5 Table: List of genes harboring mutations exclusively found in stage-1 eNSCLC patients included in cluster 0

Gene name				
ABT1	*CTNS*	*HMGB3*	*PANK2*	*ST6GALNAC4*
ACTL6A	*CUZD1*	*HOPX*	*PEA15*	*STH*
AHCYL1	*CXCL13*	*HOXB6*	*PHB2*	*STMN4*
ALOX12	*DAZAP1*	*HSD17B3*	*PHF2*	*SUCLG1*
ANKRD46	*DCSTAMP*	*IGF2-AS*	*PLA2G2A*	*SUPT7L*
APOC1	*DCUN1D4*	*KLF11*	*PRAMEF5*	*SYT15*
ARNTL	*DHX29*	*KRTAP6-2*	*PROSER2*	*TAF5*
ASAP3	*DIP2C-AS1*	*LAPTM4A*	*PRR22*	*TALDO1*
ATP6V1E2	*DNAJC17*	*LCE2A*	*PRSS54*	*TIGD6*
AZGP1	*DPH5*	*LIPN*	*PTX4*	*TM2D3*
BCL2L1	*EEF1A1*	*LRFN3*	*RAB37*	*TMCO2*
BCL2L2	*EEF1AKMT4*	*LRRC75A*	*RAB4A*	*TMEM256*
BIVM	*EFNA1*	*LSM11*	*RBM34*	*TMEM99*
C16orf82	*EFNB1*	*LYSMD4*	*RELB*	*TMX1*
C19orf48	*EIF4E3*	*MAP2*	*RETSAT*	*TSPAN17*
C1orf50	*ENAH*	*MBNL1*	*ROPN1B*	*TSTD2*
C1R	*FAM90A10P*	*MED14*	*RPL23*	*TWSG1*
C22orf23	*FAM91A1*	*MEOX1*	*S100A11*	*UBE2J2*
C9orf47	*FAM9B*	*METAP1*	*SH3BGRL*	*UBE2M*
C9orf72	*FAXDC2*	*MIR103A2*	*SKA3*	*UGT1A1*
CA1	*FOSB*	*MIR1291*	*SLC25A14*	*UGT1A7*
CALM2	*FUT2*	*MIR4713HG*	*SLC25A47*	*UPK1A*
CCK	*FXYD5*	*MIR585*	*SLC46A3*	*UQCR10*
CCL26	*GABARAP*	*MOK*	*SNAPC2*	*VPS37A*
CDC20	*GAR1*	*MREG*	*SNORD115-15*	*WNT9B*
CDC42EP1	*GFRA3*	*MROH8*	*SNORD116-14*	*XKR6*
CDK20	*GPR135*	*MRPL10*	*SNORD116-2*	*YEATS4*
CES4A	*GUK1*	*MTCH2*	*SNORD33*	*ZBTB5*
CHCHD6	*H2BC9*	*MZB1*	*SNORD82*	*ZNF18*
CHP2	*H3C8*	*NELFA*	*SNURF*	*ZNF445*
CKAP2	*HAGHL*	*NFKBIA*	*SP3*	*ZNF581*
CORO6	*HCAR1*	*NHLRC1*	*SSX5*	*ZNF654*
CRBN	*HLA-DPA1*	*OR4F17*	*ST3GAL6*	*ZRSR2*
CRYBB2	*HMGB2*			
