# Supplementary material for: GFPrint™: A machine learning tool for transforming genetic data into clinical insights
Source: PLoS One. 2024 Nov 27;19(11):e0311370. doi: 10.1371/journal.pone.0311370 (PMC11602062; doi:10.1371/journal.pone.0311370)
Supplement: S6 Table — (PDF) [file pone.0311370.s007.pdf]

S6 Table: List of genes harboring mutations exclusively found in pheochromocytoma patients included in cluster 0

Gene name				
ABCA12	*DHTKD1*	*JOSD1*	*OR51V1*	*SLC4A2*
ABCA2	*DOCK7*	*KAT6A*	*OR52I2*	*SNX19*
ABCD1	*DPP9*	*KBTBD2*	*OSBPL6*	*SOX5*
ABCD3	*DSC2*	*KCTD3*	*OTOP1*	*SPDL1*
ADAM2	*DST*	*KDM6B*	*OTOP3*	*SPEN*
ADCY10	*DUOX2*	*KIAA0319*	*PADI3*	*SPINK5*
ADGRG6	*DVL2*	*KLF12*	*PAIP1*	*SRP72*
AK9	*DYSF*	*KLHDC3*	*PAK3*	*SSX6P*
AKAP13	*ECHDC2*	*KLHL36*	*PAPOLA*	*ST6GAL2*
ALDH2	*EGLN1*	*KLRD1*	*PCK1*	*STXBP3*
ALKBH1	*EHMT1*	*KRTAP6-2*	*PGR*	*SUPT16H*
AOC3	*EIF4G3*	*KSR1*	*PHKA2*	*SYT3*
AP4E1	*ENOX2*	*LANCL1*	*PHTF2*	*SYT6*
APC	*EPC1*	*LARP4B*	*PIGO*	*TAB3*
APOL2	*EPHA3*	*LATS2*	*PKN1*	*TACC2*
ARAP3	*EPOR*	*LCT*	*PNO1*	*TAF1L*
ARHGEF39	*ERCC6L2*	*LINC00221*	*POLR2A*	*TBC1D1*
ARHGEF40	*ESX1*	*LLGL1*	*POLR3B*	*TBC1D10C*
ARID2	*F5*	*LPAR4*	*PPP1R10*	*TBP*
ARID3C	*FAM122C*	*LSM11*	*PPT2*	*TCAP*
ARL2	*FAM83D*	*LTBP1*	*PRG2*	*TDRD9*
ASB9	*FBXL20*	*MAGEA12*	*PRICKLE2*	*TEC*
ASRGL1	*FDXR*	*MAGEB1*	*PRKACB*	*TFR2*
ATP2A1	*FGF16*	*MALT1*	*PRKAR1A*	*THBS1*
ATP8B2	*FLII*	*MAML3*	*PRKD1*	*THOC1*
ATP9A	*FMO3*	*MAN1B1*	*PROM1*	*THSD7B*
ATR	*FOXA2*	*MARK4*	*PRRC2C*	*TM2D1*
ATRX	*FOXI2*	*MATR3*	*PSD4*	*TM9SF4*
BCAS1	*FOXM1*	*MBD5*	*PTPN5*	*TMEM208*
BCL2L2	*FZD8*	*MCOLN2*	*PVALB*	*TMEM8B*
BCR	*GATAD2B*	*MERTK*	*PYGB*	*TNKS*
BICD1	*GBP6*	*MIR4436A*	*QPCT*	*TOX3*
BPIFB6	*GJA5*	*MIR937*	*R3HDML*	*TPCN1*
BRWD3	*GLB1L3*	*MLXIP*	*RAB35*	*TPO*
BTBD10	*GLOD4*	*MMP2*	*RABEPK*	*TRIM17*
BZW2	*GOLGA4*	*MMRN1*	*RAD50*	*TRIP11*

Gene name				
C5	*GOLM2*	*MRC2*	*REPIN1*	*TRIP6*
CACYBP	*GOSR2*	*MRGPRX3*	*ROCK2*	*TRRAP*
CALCRL	*GP2*	*MSLN*	*RP1L1*	*TUB*
CASP2	*GPR137*	*MUC5AC*	*RPS27A*	*TXNRD1*
CCDC110	*GPR148*	*MYH15*	*RPS6KA5*	*UBN1*
CCDC171	*GPR156*	*MYO5C*	*RPS6KC1*	*UNC5A*
CCDC60	*GPX4*	*MYPN*	*RRBP1*	*URM1*
CCL13	*GTF2H2*	*NAV3*	*RSF1*	*USP19*
CDH20	*GULP1*	*NBN*	*RTL9*	*VAV1*
CDH5	*H1-0*	*NCAPD3*	*RTP4*	*VPS13C*
CDK5RAP1	*HAPLN4*	*NEK4*	*RWDD4*	*WDR62*
CDYL2	*HAT1*	*NFIC*	*SALL1*	*WDR64*
CELSR3	*HAVCR1*	*NIBAN1*	*SCN5A*	*WNT3*
CEP250	*HDHD5*	*NLRP4*	*SCRIB*	*WT1*
CFH	*HECA*	*NOL4*	*SEC14L3*	*XPNPEP2*
CHST15	*HECTD1*	*NOP58*	*SEC14L5*	*YME1L1*
CLASP2	*HELZ*	*NR1H4*	*SH3BP4*	*ZACN*
COASY	*HFM1*	*NRROS*	*SHB*	*ZBTB14*
COL12A1	*HSP90AA1*	*NSD2*	*SHISA3*	*ZNF275*
CPSF4	*IGLL1*	*NUCKS1*	*SHPRH*	*ZNF507*
CRAMP1	*IL15RA*	*NUGGC*	*SIMC1*	*ZNF516*
CSH2	*IL34*	*NUP98*	*SLC12A4*	*ZNF613*
CTH	*IQSEC3*	*NUTM1*	*SLC12A8*	*ZNF676*
CYP2B6	*IRF9*	*OLIG3*	*SLC26A9*	*ZNF687*
CYP4F2	*ITLN2*	*OPRPN*	*SLC35C1*	*ZNF799*
DCAF11	*IVL*	*OR10T2*	*SLC38A4*	*ZZEF1*
DGAT2	*IWS1*	*OR10X1*		
