# Supplementary material for: GFPrint™: A machine learning tool for transforming genetic data into clinical insights
Source: PLoS One. 2024 Nov 27;19(11):e0311370. doi: 10.1371/journal.pone.0311370 (PMC11602062; doi:10.1371/journal.pone.0311370)
Supplement: S7 Table — (PDF) [file pone.0311370.s008.pdf]

S7 Table: List of 75 genes belonging to the PI3K/Akt signaling pathway that were selected from breast cancer patients included in SP1 after the triage described in the text.

Gene name				
ANGPT4	*CRTC2*	*IRS1*	*LAMB4*	*PPP2R5B*
ATF2	*CSF1*	*ITGA1*	*LAMC1*	*PPP2R5C*
CCNE2	*EFNA3*	*ITGA11*	*LAMC2*	*PPP2R5D*
COL1A2	*EFNA5*	*ITGA7*	*LAMC3*	*PRLR*
COL4A1	*FGF18*	*ITGA9*	*LPAR1*	*RELN*
COL4A2	*FGF6*	*ITGB4*	*LPAR5*	*RHEB*
COL4A3	*FGF8*	*ITGB5*	*MAGI1*	*RPS6*
COL4A4	*FN1*	*ITGB6*	*PCK1*	*RPS6KB2*
COL4A6	*GNB3*	*LAMA1*	*PCK2*	*SGK2*
COL6A2	*GNB5*	*LAMA2*	*PDGFC*	*SPP1*
COL6A3	*GYS1*	*LAMA3*	*PHLPP1*	*THBS3*
COL9A1	*HGF*	*LAMA4*	*PHLPP2*	*TNN*
COL9A3	*IFNA16*	*LAMA5*	*PPP2CB*	*TNXB*
COMP	*IFNA2*	*LAMB1*	*PPP2R2B*	*VEGFD*
CREB3L3	*IFNA21*	*LAMB2*	*PPP2R3C*	*YWHAB*
